# Research to Reality: Moving Evidence Into Practice Through an Online Community of Practice

**DOI:** 10.5888/pcd11.130272

**Published:** 2014-05-08

**Authors:** Margaret M. Farrell, Madeline La Porta, Alissa Gallagher, Cynthia Vinson, Sarah Bruce Bernal

**Affiliations:** Author Affiliations: Madeline La Porta, Alissa Gallagher, Cynthia Vinson, National Cancer Institute, Bethesda, Maryland; Sarah Bruce Bernal, ICF International, Fairfax, Virginia.

## Abstract

How can a community of practice help further the practical application of cancer control research? In 2011, the National Cancer Institute (NCI) launched an online community of practice, Research to Reality (R2R). R2R aims to infuse evidence-based strategies into communities by engaging researchers and practitioners in a joint approach to research dissemination. To measure community growth and engagement, NCI measures data across 3 program domains: content, interaction, and activity. NCI uses Web analytics, usability testing, and content analyses to manage and evaluate R2R. As of December 2013, R2R had more than 1,700 registered members. More than 500 researchers and practitioners register for the monthly cyber-seminars, and 40% return each month. R2R hosts more than 15,500 page views and 5,000 site visits in an average month. This article describes the process of convening this online community and quantifies our experiences to date.

## Introduction

Dissemination, the targeted distribution of information and intervention materials to a specific public health or clinical practice audience, is a critical aspect of cancer control practice. Dissemination strategies for evidence-based interventions aim to spread knowledge and interventions on a wide scale in or across geographic locations, practice settings, or social or other networks of end users such as patients and health care providers (1). Traditional dissemination strategies (ie, publishing in peer-reviewed journals; presenting at professional conferences; and academic detailing, a form of professional outreach) are essential but not sufficient to inform cancer control practice. Translating research into everyday practice is a critical problem in both clinical and public health arenas, and the goal of implementing scientific evidence into practice remains unmet (2–5).

In general, researchers in academia are responsible for developing and conducting research studies that generate evidence, and clinicians and practitioners are responsible for adapting evidence into everyday practice (6). Therefore, building authentic partnerships between researchers and practitioners is central to successful translation efforts (7,8). Strong community partnerships provide access to populations and engender the trust necessary to implement evidence-based interventions. However, the practical application of research remains challenging for both researchers and practitioners. Community engagement has been largely successful in bridging the gap between researchers and community practitioners (9,10). A forum is needed to allow both to engage in an ongoing dialogue about their mutual goal of improving lives by putting research into practice in the field.

## Bridging the Gap Between Researchers and Community Practitioners

To further the practical application of evidence-based cancer control practice, the National Cancer Institute (NCI) created the Cancer Information Service (CIS) Partnership Program in 1993. The regionally based national program formed academic and community partnerships with more than 900 organizations and coalitions. By working directly with researchers and practitioners, the CIS Partnership Program built partners’ capacity to identify evidence-based interventions, adapt them to their communities, and disseminate programs that addressed cancer health disparities (11). Central to this work was providing partners with technical assistance and training on the use of evidence-based planning tools such as NCI’s Cancer Control P.L.A.N.E.T. (Plan, Link, Act, Network with Evidence-based Tools; http://ccplanet.cancer.gov), a Web portal that provides cancer control practitioners a process for accessing data, partners, programs, and resources to assist in planning evidence-based programs (12).

When the program ended in January 2010, NCI looked for other ways to support research dissemination activities and collaborations established through the CIS Partnership Program. The national network of regionally based CIS staff perceived great benefit in their ability to engage not only with NCI, but also with one another to share experiences, tools, and resources. In this way, the CIS evolved from a network into an informal community of practice, which exemplifies social learning theory in that it uses engagement as the fundamental process to share knowledge (13). Communities of practice are groups of people who share a concern, set of problems, or passion about a topic and who deepen their knowledge and expertise in this area by interacting on an ongoing basis.

While rapidly growing social media use by NCI and other government agencies created an ideal opportunity to create a Web-based dissemination program, the emergence of topic-based online communities of practice showed increasing promise (14). Such an approach would continue the work of the CIS Partnership Program in building community capacity and translating evidence-based cancer control research into practice. It would also provide an opportunity for researchers and practitioners to regularly engage with one another.

In 2009, Cancer Control P.L.A.N.E.T. was enhanced and revised. NCI queried CIS partners and researchers to test the idea of creating an online community of practice to address challenges of research dissemination and implementation. CIS partners and researchers whose work was included in P.L.A.N.E.T.’s Research-Tested Intervention Programs (RTIPs) repository were asked what the term *community of practice* meant to them. Respondents suggested enhancing Cancer Control P.L.A.N.E.T. to include interactive features such as an online forum or a discussion board, a knowledge repository to store conversations, mentorship opportunities, researcher interactions, online training, and user-generated content. Details about the survey and results are reported elsewhere (15).

In 2010, these factors converged, and the result was a concept to develop Research to Reality (R2R) (https://researchtoreality.cancer.gov) as an online community of practice, bringing researchers and practitioners together for the purpose of building capacity through a peer-to-peer learning or apprenticeship model, such as those described in the community of practice literature (16).

## Conceptual Framework for Research to Reality

**Figure 1:**
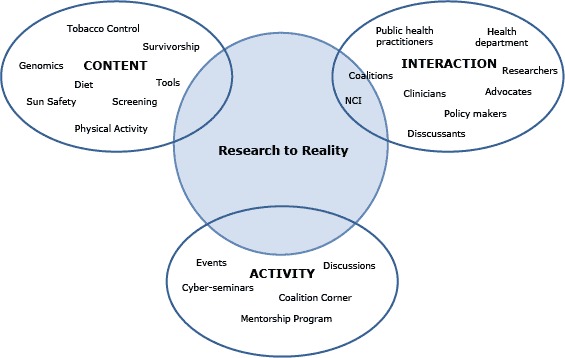
Visualizing a virtual community of practice to move cancer control research into practice, Research to Reality, National Cancer Institute, 2011.

At its inception, R2R sought to integrate these 3 concepts: the success of the CIS Partnership Program model in engaging cancer control researchers and practitioners, community of practice theory, and the increasing use of social media in all areas of the government and private sectors. In creating the conceptual framework for the R2R community of practice (Figure 1), NCI drew heavily from Wenger’s Participatory Framework Model (13), adapting it to reflect the successful aspects of the CIS Partnership Program and to address the previously identified gap between cancer control science and practice. To this end, NCI focused its work on developing the R2R Web platform around 3 community dimensions: content, interaction, and activity.

From its outset, R2R sought to: 1) create an online community of practice for cancer control practitioners and researchers, 2) engage cancer control practitioners and researchers with all levels of experience in an ongoing dialogue around moving research into practice (interaction), and 3) build capacity and help foster collaborations in the cancer control field by highlighting successful evidence-based interventions and programs (content) and offering dynamic communication, learning, and mentorship opportunities (activity).

To begin convening researchers and practitioners while the platform was built and to begin developing a sense of community identity and purpose, NCI launched a series of monthly cyber-seminars. These Web-based presentations were unique in their approach; they focused on translating research to practice, fostering engagement and dialogue by pairing researchers with practitioners. The cyber-seminars provided a forum to discuss successes and challenges around implementing evidence-based cancer control programs, set the tone for R2R dialogue, and established the community’s purpose. The first R2R cyber-seminar was conducted in January 2010 with more than 1,100 registrants and 700 participants. Since the first webinar through December 2013, nearly 20,000 people have registered for the yearly, 10-month cyber-seminar schedule. On average, R2R sees 675 registrants and 260 participants each month. These monthly cyber-seminars continue and are an essential aspect of the community’s work to provide content, interaction, and an opportunity for capacity building.

## Developing an Online Community of Practice in the Federal Space: Building Research to Reality

NCI held a series of requirements-gathering sessions, which helped identify a Drupal-based (https://drupal.org/) platform that possessed the functionality and features necessary to implement the R2R model. This cost-effective platform could be implemented quickly and allows for iterative design and development through its large developer network and add-on modules.

The decision to establish a federal government–sponsored community of practice created unique programmatic considerations. Meeting federal accessibility, privacy, and security requirements posed significant challenges. Creating a community identity and building trust through the sharing of personally identifiable information (name, organization, research interests, and photos) among registered members was viewed as critical to the success of R2R but raised bureaucratic scrutiny. User testing indicated that these compromises were offset by the perceived benefit of and the opportunity to highlight their work on an NCI site.

In February 2011, NCI launched the website with initial features that included discussions, webinars, featured partners, and events. Exploration of additional content, activity, and interaction strategies continued as R2R evolved. One such expansion was the R2R Mentorship Program. This pilot program was designed to build the capacity of cancer control practitioners to effectively navigate the complex, “real world” context (eg, inadequate resources, political barriers, organizational constraints) in which evidence-based decision making occurs. Through the website, R2R community members followed 6 mentor-mentee pairs through monthly storyboards that highlighted their progress as they implemented evidence-based programs in their communities (17). Community members asked questions of the mentors and mentees and in several cases added their own encouragement and reflections. The first set of mentee-mentor pairings has concluded, and a collection of articles of their work are in press with *Preventing Chronic Disease*.

NCI actively seeks “strategic content providers” to foster collaborations and enhance interactions between and among researchers and practitioners. The addition of a recurring Coalition Corner discussion in 2012 furthered NCI’s collaboration with the Comprehensive Cancer Control National Partnership (18). Coalition Corner provides cancer control coalitions in the states, tribal nations, and territories a virtual space for asking questions, discussing achievements, and sharing challenges. R2R has featured 34 contributors, spawning discussions on the platform and requests from R2R members for related cyber-seminars to further explore the issues.

Similarly, R2R established a “virtual cross-walk” with P.L.A.N.E.T.’s RTIPs repository. As a new program is added to the repository, the lead researcher engages community members in a discussion about adapting the intervention for their own communities. In collaboration with the NCI’s Implementation Science team, R2R provides an online discussion platform and archive for the monthly Advanced Topics in Implementation Science webinar series. This series convenes scores of implementation scientists monthly to share perspectives on current topics and their work.

## Evaluating Research to Reality

The ongoing, systematic evaluation of R2R has been essential for its content management and community development. Although R2R is a Web-based platform, measuring and tracking Web analytics alone would not capture the growth, development, or vitality of the community of practice. As such, NCI adapted the Macuarium Set of Community Practice Measurements (19) and collects data to measure each of the community of practice dimensions.

In 2013, the R2R website had 80,000 page views with an average monthly page view of 3,200. The home page and the cyber-seminars make up the most visited sections of the site, followed by discussions and the featured partners pages. Registration and membership data are also tracked monthly, and these numbers have grown steadily. During 2012–2013, approximately 1,750 new users joined R2R. The 41 cyber-seminar sessions held through June 2013 attracted more than 32,000 registrants and nearly 11,000 participants. These data demonstrate that the R2R cyber-seminar series fills a need for members of our target audience and suggests that they are not getting this content from other sources.

**Figure 2:**
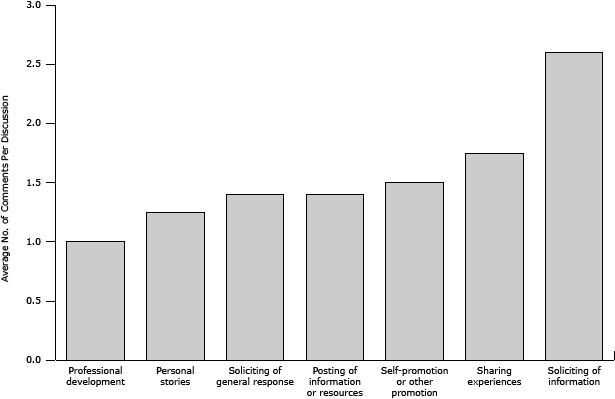
Average comments generated in response to different discussion types, Research to Reality, National Cancer Institute, 2013. Data source: Researchtoreality.cancer.gov (20). **Table:** 

A mixed-method content analysis using qualitative research analysis software (http://www.dedoose.com/) was used to evaluate the level of engagement with R2R discussions and the types of content or cues that prompted R2R members to post and interact with one another. Findings showed that the most commonly posted community generated cancer-issue posts were around communication and program implementation and dissemination. R2R members were more likely to respond to posts when a colleague was asking for help or advice (Figure 2), consistent with the literature on community engagement (21). The NCI uses a “Contact Us” feature and regular member surveys to collect user feedback. Data derived from these sources are indicators of community engagement and are used for continued quality improvement.

Comment Topic	Average No. of Comments Per Discussion
Professional development	1.0
Personal stories	1.25
Soliciting of general response	1.4
Posting of information or resources	1.4
Self-promotion or other promotion	1.5
Sharing experiences	1.75
Soliciting of information	2.6

As the community has grown, determined on the basis of these indicators, so has the level of engagement on the site. Program data demonstrate a 62% increase in community-generated discussions between 2011 and 2013. The number of community-initiated discussions, however, does not yet meet NCI’s program expectations. Through programmatic efforts and further community development, the goal is to have R2R community members generate closer to half of the content on the site.

## Discussion

Communities of practice, particularly in the virtual space, are increasingly being used by government agencies to share knowledge, tackle problems, and interact with partners, grantees, and the public across geographic locations. NCI developed R2R with specific goals in mind: to engage practitioners and researchers in an ongoing dialogue, to build capacity for evidence-based program planning, and to foster collaborations that address the problem of dissemination and implementation. NCI imagined that a virtual community of practice could facilitate the authentic engagement of researchers and practitioners necessary to move evidence-based programs into action.

R2R has been successful in many ways. The community has attracted a robust membership from many disciplines. Practitioners and researchers regularly join monthly cyber-seminars and are eager to showcase their work in that forum. Anecdotal stories and discussions posted on R2R demonstrate its potential to attract the right members and conduct capacity-building activities despite a dwindling financial climate.

Engagement, however, is what distinguishes a community of practice from a static website. Although NCI has successfully generated discussions on cyber-seminars, fewer discussions originate unsolicited online. Few published benchmarks identify measures of success for virtual communities of practice. Further evaluation is needed to better understand how to leverage the current site traffic to drive community engagement. R2R is a promising strategy to address a longstanding problem and is well-positioned to advance understanding of the potential roles of virtual communities of practice for improving cancer control practice.
